# Morning glory disc anomaly associated with large facial infantile hemangioma as the presenting signs of PHACE syndrome

**DOI:** 10.5935/0004-2749.20200071

**Published:** 2020

**Authors:** Kenzo Hokazono, Vinícius Tadashi Okuyama, Leonardo Provetti Cunha, Mário Luiz Ribeiro Monteiro

**Affiliations:** 1 Division of Ophthalmology, Faculdade de Medicina, Universidade de São Paulo, São Paulo, SP, Brazil; 2 Department of Ophthalmology, Universidade Federal do Paraná, Curitiba, PR, Brazil; 3 Universidade Federal de Juiz de Fora, Juiz de Fora, MG, Brazil

**Keywords:** Hemangioma, Eye abnormalities, Aortic coarctation, Neurocutaneous syndrome, Magnetic resonance imaging, Humans, Case reports, Hemangioma, Anormalidades do olho, Coartação aórtica, Síndromes neurocutâneas, Imagem por ressonância magnética, Humanos, Relatos de casos

## Abstract

Infantile hemangioma, the most common benign tumor in infancy, is usually an isolated condition occurring in many different locations in the body. However, large infantile hemangioma may be associated with other systemic malformations, including central nervous system, cerebrovascular, cardiac, and ophthalmology abnormalities, a condition termed *PHACE syndrome*. In this paper, we describe a case of PHACE syndrome that was presented with the unique association of a large facial infantile hemangioma and morning glory anomaly.

## Abstract (pt)

O hemangioma infantil é a causa mais comum de tumor benigno na infância e usualmente é uma condição isolada podendo ocorrer em diferentes regiões do corpo. No entanto, hemangiomas infantil extensos podem ser associados com outras malformações sistêmicas incluindo anomalias no sistema nervoso central, cerebrovasculares, cardíacas e oftalmológicas, uma condição denominada síndrome PHACE. Neste trabalho, descrevemos o caso de um paciente com síndrome PHACE que se apresentou com um extenso hemangioma facial e anomalia de “morning glory”.

## INTRODUCTION

Morning glory disc anomaly (MGDA) is a rare congenital anomaly typically affecting the unilateral optic disc. It is characterized by an enlarged funnel-shaped excavation in the optic disc, an annulus of chorioretinal pigmentary abnormalities that surrounds the optic disc, a central glial tuft overlying the optic disc, and a distribution pattern of retinal blood vessels that originates at the disc margin with a straight and radial orientation^(1)^. Although MGDA anomaly can be an isolated finding, it may also be associated with a number of cranial anomalies such as hypertelorism, basal encephalocele, cleft lip, agenesis of the corpus callosum, and Moyamoya disease^(2)^. PHACE syndrome is an acronym for *p*osterior fossa malformations, *h*emangioma, *a*rterial anomalies, *c*oarctation of the aorta/cardiac defects, and eye abnormalities^(3)^. The clinical hallmark of PHACE syndrome is a large and segmental infantile hemangioma (IH) usually present on the face, neck, and/or scalp region. We described a unique case of MGDA associated with a large facial IH as part of PHACE syndrome.

## CASE REPORT

**Figure 1:**
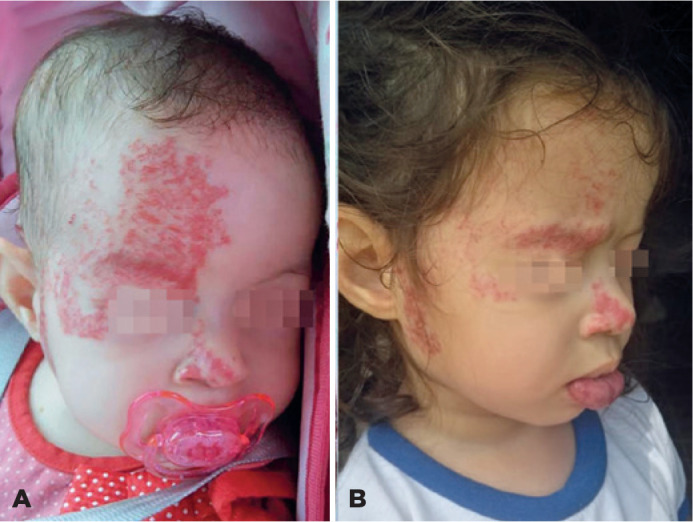
Extensive hemangiomas on the right side of the face. A) Large hemangiomas at the beginning of the treatment. B) Hemangiomas after treatment was consolidated.

**Figure 2:**
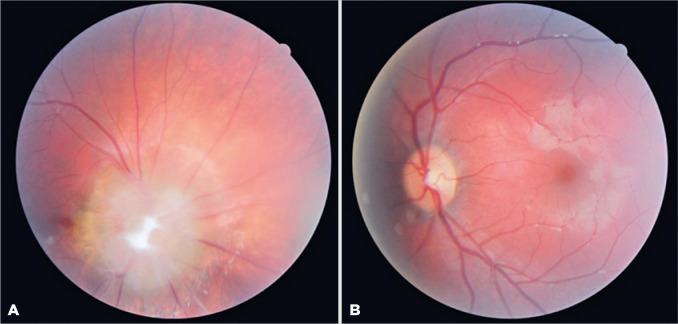
A) Fundus photograph showing morning glory anomaly in the right eye. B) Normal fundus appearance in the left eye.

A 1-year-old girl with an extensive facial hemangioma noted by the family in the second week of life, which increased in size progressively, was referred for ophthalmologic evaluation. On examination, she had a large hemangioma in the right side of the face, involving the frontotemporal, periorbital, and periauricular region (Figure 1A). Ocular motility, biomicroscopy, and tonometry were normal. Nevertheless, pupil examination revealed a relative afferent pupillary defect in the right eye. Fundoscopic examination demonstrated an MGDA in the right eye (Figure 2) and was normal in the left eye.

**Figure 3:**
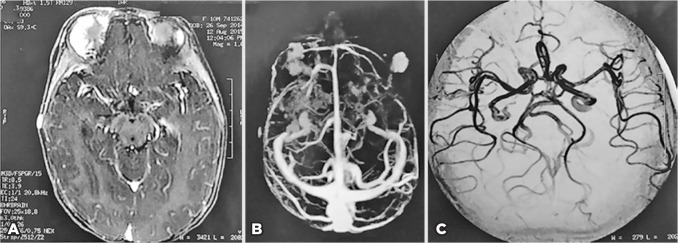
Magnetic resonance angiography of the brain and face. A) Axial image showing the right periorbital hemangioma. B) Multiples areas of pathological contrast enhancement suggestive of hemangiomas. C) Willis polygon without abnormalities.

Considering those clinical findings, a diagnosis of PHACE syndrome was made (hemangioma greater than 5 cm plus MGDA). In this way, screening to detect other malformations was performed. Magnetic resonance angiography of the brain and face showed multiple areas of pathological contrast enhancement suggestive of superficial frontotemporal hemangiomas on the right side and a malar on the left side, but cerebral vascular disorders and malformations were not observed (Figure 3). No abnormalities were seen on echocardiogram, abdominal Doppler ultrasound, or electroencephalography. The systemic clinical assessment was normal, except for a glottic hemangioma in the otolaryngologic examination.

The patient was treated with propranolol (2 mg/kg/day), and an escalating drug regimen was adopted by the pediatric service. At the patient’s last follow-up (1 year after the initial visit), no side effects of the drugs were observed, and the size of the facial hemangioma had decreased (Figure 1B).

## DISCUSSION

Infantile hemangioma is a common benign tumor in children often presenting as an isolated finding. However, in cases with large (>5 cm) and segmental (covering an anatomic territory of the face or body) IH, particularly in the scalp, face, and neck region, a thorough workup is required to detect other abnormalities associated with PHACE syndrome. This syndrome was described in 1996 by Frieden et al.^(3)^, and the diagnostic criteria were revised in 2016 (Table 1)^(4)^. Our patient presented with IH (>5 cm) on the face associated with one major criterion (i.e., MGDA), defining the diagnosis of PHACE syndrome. It is worth noting that MGDA is usually associated with transsphenoidal encephalocele, requiring workup for hypopituitarism. Our case is interesting because the identification of MGDA helped to establish the diagnosis of PHACE syndrome. Defining the association is extremely important because in PHACE syndrome, other vascular anomalies must be investigated as intracranial vascular malformations and cardiovascular anomalies have a great potential to cause long-term morbidity. Children with PHACE syndrome have a risk of arterial ischemic stroke (AIS) due to limitations of blood flow and steno-occlusive disease of the main cerebral vessels at or above the circle of Willis. Sigel et al.^(5)^ suggested that aplasia, hypoplasia, or occlusion of a major cerebral artery are significant risk factors for AIS in patients with PHACE syndrome. In addition, the prevalence of congenital heart disease, mainly aorta coarctation, in patients with PHACE syndrome ranges from 41% to 67%^(6)^. In contrast to typical aortic arch anomaly, which is characterized by juxtaductal narrowing on the ascendant aorta segment, patients with PHACE syndrome have complex involvement and extensive narrowing of the transverse and descendent segments of the aortic arch. Fortunately, our case had a negative screening for other cerebrovascular and cardiac anomalies, with only an airway hemangioma located at the glottis, which did not cause any clinical symptoms^(6)^.

**Table 1:** Diagnostic criteria for PHACE syndrome based on the consensus from 2016.

Organ Systems	Major Criteria	Minor Criteria
Arterial anomalies	Anomaly of major cerebral or cervical arteries; dysplasia of the large cerebral arteries; arterial stenosis or occlusion; absence or moderate-severe hypoplasia of the large cerebral and cervical arteries; aberrant origin or course of the large cerebral or cervical arteries; persistent carotid-vertebrobasilar anastomosis	Aneurysm of any of the cerebral arteries
Structural brain	Posterior fossa brain anomalies; Dandy-Walker complex; other hypoplasia/dysplasia of the mid and/or hind brain	Midline brain anomalies Malformation of cortical development
Cardiovascular	Aortic arch anomalies; coarctation of the aorta; dysplasia; aneurysm; aberrant origin of the subclavian artery	Ventricular septal defect Right aortic arch/double aortic arch Systemic venous anomalies
Ocular	Posterior segment abnormalities; persistent hyperplastic primary vitreous; persistent fetal vasculature; retinal vascular anomalies; morning glory disc anomaly; optic nerve hypoplasia; peripapillary staphyloma	Anterior segment abnormalities; microphthalmia; sclerocornea; coloboma; cataracts
Ventral/midline	Anomaly of the midline chest and abdomen - Sternal defect; sternal pit; sternal cleft; supraumbilical raphe	Ectopic thyroid hypopituitarism Midline sternal papule/hamartoma
	**Definite PHACE**	**Possible PHACE**
Hemangioma >5 cm of the head, including scalp	+1 major or 2 minor criteria	+1 minor criteria
Hemangioma of the neck, upper trunk, or trunk and proximal upper extremity	+2 major criteria	+1 major or 2 minor
No hemangioma		+2 major criteria

Garzon MC, Epstein LG, Heyer GL, Frommer PC, Orbach DB, Baylis AL, et al. PHACE Syndrome: Consensus-Derived Diagnosis and Care Recommendations. J Pediatr. 2016;178:24-33. e22.^(4)^

PHACE syndrome may be associated with other neurologic and vascular malformations that could be correlated with the same embryologic origin, which suggests that this syndrome might have a variably expressed spectrum or an overlap between malformations^(7)^.

In our patient, the facial hemangioma was treated with systemic propranolol for 1 year with important regression of the facial lesion, confirming previous studies that document the role of such treatment for IH^(8)^. It is important to keep in mind other long-term morbidities associated with PHACE syndrome, such as hearing loss due to intracranial hemangiomas involving auditory structures^(9)^, dysphagia, or speech disorders associated with airway hemangiomas^(10)^, and headaches, which are more prevalent in PHACE syndrome patients. In addition, patients with major criteria without large IH should be investigated for the presence of extracutaneous hemangiomas such as intraorbital/periorbital, intestinal tract, and airway IH^(10)^.

In conclusion, our case serves to document that PHACE syndrome may include the combination of large IH and MGDA. Awareness of such an association is important to establish the correct diagnosis of PHACE syndrome and help determine the correct investigation and treatment of other potentially life-threatening abnormalities that may be associated with this condition.
